# *FUT2* Secretor Status Influences Susceptibility to VP4 Strain-Specific Rotavirus Infections in South African Children

**DOI:** 10.3390/pathogens9100795

**Published:** 2020-09-27

**Authors:** Jaime MacDonald, Michelle J. Groome, Janet Mans, Nicola Page

**Affiliations:** 1 National Institute for Communicable Diseases, Sandringham 2131, South Africa; nicolap@nicd.ac.za; 2 Department of Medical Virology, Faculty of Health Sciences, University of Pretoria, Pretoria 0001, South Africa; janet.mans@up.ac.za; 3 South African Medical Research Council, Vaccines and Infectious Diseases Analytics Research Unit, Faculty of Health Sciences, University of Witwatersrand, Johannesburg 2193, South Africa; groomem@rmpru.co.za

**Keywords:** rotavirus, secretor status, histo-blood group antigens, VP4 genotypes, *FUT2*, susceptibility, vaccines

## Abstract

Gastroenteritis is a preventable cause of morbidity and mortality worldwide. Rotavirus vaccination has significantly reduced the disease burden, but the sub-optimal vaccine efficacy observed in low-income regions needs improvement. Rotavirus VP4 ‘spike’ proteins interact with FUT2-defined, human histo-blood group antigens on mucosal surfaces, potentially influencing strain circulation and the efficacy of P[8]-based rotavirus vaccines. Secretor status was investigated in 500 children <5 years-old hospitalised with diarrhoea, including 250 previously genotyped rotavirus-positive cases (P[8] = 124, P[4] = 86, and P[6] = 40), and 250 rotavirus-negative controls. Secretor status genotyping detected the globally prevalent G428A single nucleotide polymorphism (SNP) and was confirmed by Sanger sequencing in 10% of participants. The proportions of secretors in rotavirus-positive cases (74%) were significantly higher than in the rotavirus-negative controls (58%; *p* < 0.001). The rotavirus genotypes P[8] and P[4] were observed at significantly higher proportions in secretors (78%) than in non-secretors (22%), contrasting with P[6] genotypes with similar proportions amongst secretors (53%) and non-secretors (47%; *p* = 0.001). This suggests that rotavirus interacts with secretors and non-secretors in a VP4 strain-specific manner; thus, secretor status may partially influence rotavirus VP4 wild-type circulation and P[8] rotavirus vaccine efficacy. The study detected a mutation (rs1800025) ~50 bp downstream of the G428A SNP that would overestimate non-secretors in African populations when using the TaqMan® SNP Genotyping Assay.

## 1. Introduction

Gastroenteritis is a preventable cause of morbidity and mortality worldwide, and the burden predominantly exists in high-risk populations such as children under the age of five years in low-income regions [1]. Rotavirus is the most frequent aetiology of diarrhoeal illness and death in children <5 years-old, and it was responsible for 29% of global diarrhoeal deaths occurring in this age group in 2016 [2]. 

The introduction of oral rotavirus vaccines in >100 countries worldwide has significantly reduced the burden of rotavirus diarrhoea and resulted in a 38% overall reduction in childhood diarrhoeal hospitalisations globally [3,4]. However, rotavirus vaccine efficacy appears to vary significantly between high-income (85–98%) and low-income (50–64%) countries [5]. Eliciting an adequate immune response to oral vaccines is multifactorial but may be limited in low-income settings due to impoverished living conditions and increased exposure to pathogens [3]. In addition, the passive transfer of rotavirus maternal antibodies during breastfeeding can influence the immune response elicited by oral rotavirus vaccines in young children [4]. Understanding the factors that have contributed to an observed lower rotavirus vaccine efficacy in these settings may alleviate the burden of rotavirus-associated mortality in children. 

Host genetic factors have recently been proposed to influence susceptibility to enteric pathogens. The excretion of soluble human histo-blood group antigen (HBGA) structures in gut mucosal surfaces determines a host’s ‘secretor status,’ controlled by the human *FUT2* gene. Non-secretor phenotypes with an inability to express soluble HBGAs due to mutations in the *FUT2* gene (such as the prevalent G428A SNP; rs601338) are present globally in varying proportions. Higher proportions of non-secretor phenotypes are observed in African populations (~30%) than in Asian populations (~5%) [6,7]. 

Antigenic HBGA structures present in the body can act as receptors for various pathogens to bind during infection [8,9]. *FUT2* secretor status can modulate infection because it defines the presence (secretor) or absence (non-secretor) of HBGA attachment factors excreted in the gut. Susceptibility to enteric norovirus infection has been associated with secretor status, where non-secretor phenotypes have been found to display a natural resistance to GII.4 norovirus strains [10,11,12]. It has been proposed that variations in secretor status phenotypes and subsequent differences in host-defined susceptibility may contribute to the circulation of rotavirus strains in a similar mechanism [13]. 

Interactions between rotavirus particles and HBGA receptors present in the gut can occur via the VP4 (VP8* subunit) ‘spike’ protein on the surface of the virion [14]. Evidence of rotavirus VP4 strain-specific binding patterns between HBGAs and prevalent strains (P[8], P[4], and P[6]) has recently been noted [14]. Rotavirus P-types have distinct VP4 morphology that determines the presence or absence of HBGA-binding interfaces, allowing for different mechanisms of binding and entry of rotavirus particles to occur [13]. Studies have shown that rotavirus genotypes P[8]- and P[4]-bound complex and soluble HBGAs abundant in secretors, as well as an increased susceptibility to infection with these rotavirus strains in secretors. Non-secretors with an absence of HBGAs in the gut have been found to display a natural resistance to P[8] and P[4] strains with VP4 HBGA-binding interfaces [15,16,17]. Variations in host-defined secretor status can therefore influence susceptibility to infection with different rotavirus strains. 

Rotavirus P[8] genotypes are responsible for more than 80% of human wild-type infections globally [15]. However, rotavirus circulation in Africa differs in strain diversity and prevalence, with more frequent cases of P[6] strains, which have reached 26% of all rotavirus strains circulating in African populations [18]. The proportions of naturally resistant non-secretors may alter the circulation of rotavirus P-types compared to that in global populations. 

The Rotarix^®^ and RotaTeq^®^ rotavirus vaccines both contain P[8]-based strains or reassortants, and they provide protection through the replication of live-attenuated vaccine strains in the gut to induce a local immune response [4]. Associations between host-defined secretor status and susceptibility to infection with specific rotavirus strains pose interesting questions surrounding the lowered efficacy of P[8]-based rotavirus vaccines observed in some regions [19,20]. Emerging research has alluded to this idea [21,22,23], including the influence of the related *FUT3* Lewis host genetic factor [24,25,26,27], but further investigations are required. These data have contributed to the evidence that host genetic factors such as secretor status can influence infections by pathogens including rotavirus, as well as that strain-specific interaction mechanisms may occur [14,15,28]. 

The aim of this study was to investigate *FUT2*-defined secretor status in South African children <5 years-old hospitalised with diarrhoea and to examine the association between a host’s genetic secretor status and rotavirus-associated hospitalisations. Understanding the relationship between pathogens such as rotavirus and the genetics of a population may identify avenues for improvements in vaccine efficacy to reduce the burden of rotavirus gastroenteritis.

## 2. Results

Secretor genotypes were successfully determined for all 500 children selected for the study, and the total cohort comprised 65.8% (329) secretors with at least one functional *FUT2* allele and 34.2% (171) non-secretors with both *FUT2* alleles containing the G428A SNP. 

Rotavirus-positive cases (RV+) comprised 74% (185/250) secretors (Se) and 26% (65/250) non-secretors, while rotavirus-negative controls (RV-) comprised 58% (144/250) secretors and 42% (106/250) non-secretors. The distributions of secretors versus non-secretors observed amongst cases and controls were significantly different (*p* < 0.001). 

Information on rotavirus genotyping from the Rotavirus Sentinel Surveillance Program (RSSP) database [29,30] showed that the rotavirus-positive cases (n = 250) comprised 124 P[8] infections, 86 P[4] infections, and 40 P[6] infections (Supplementary Material). The proportions of secretors and non-secretors were compared amongst each VP4 strain within rotavirus-positive cases (Table 1). Rotavirus P[8] infections (79% secretors and 21% non-secretors) and P[4] infections (77% secretors and 23% non-secretors) had significantly different proportions of secretor phenotypes compared to P[6] infections (53% secretors and 47% non-secretors) (*p* = 0.001 and *p* = 0.006, respectively). When considered together, rotavirus P[8] and P[4] infections (78% secretors and 22% non-secretors) had significantly different proportions of secretor phenotypes compared to P[6] infections (53% secretors and 47% non-secretors) (*p* = 0.001). 

**Figure 1:**
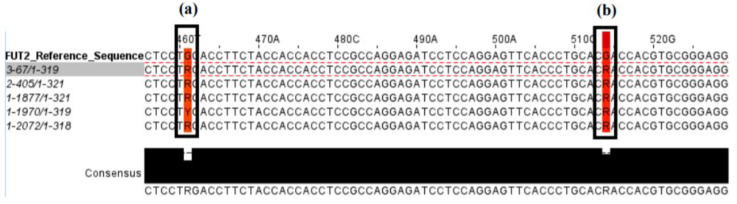
Sequence alignment of five participants where Sanger sequencing and RT-PCR genotyping results were discrepant. (**a**) The G428A SNP location displaying all discrepant sequences containing the two peaks ‘G’ and ‘A,’ as represented by an ‘R’ annotation. (**b**) The mutation site (rs1800025) located ~50 base pairs downstream of the G428A SNP, common in all discrepant results.

The Sanger sequencing of the exon 2 region of the *FUT2* gene conducted for 10% of the cohort confirmed the presence of either functional *FUT2* alleles or G428A SNP alleles for 91% (48/53) of analysed specimens. Sequences of the *FUT2* exon 2 region from 12 homozygous secretors (SeSe), 24 heterozygous secretors (Sese), and 17 homozygous non-secretors (sese) were obtained and compared to RT-PCR G428A genotyping results. Five discrepant results were observed in which heterozygous secretor (Sese) individuals (one functional *FUT2* allele and one allele containing the non-functional G428A SNP) genotyped by Sanger sequencing were incorrectly genotyped by RT-PCR as non-secretors (both alleles containing the G428A SNP). A commonality between these discrepant specimens was an SNP mutation (rs1800025) ~50 bp downstream of the G428A SNP (Figure 1).

## 3. Discussion

The results from this study indicate that secretors were more susceptible to rotavirus infection, and non-secretors seemed to display a natural resistance. The absence of HBGAs in the gastric mucosa of non-secretors appeared to reduce susceptibility to rotavirus, possibly by limiting the attachment stage of binding and entry during rotavirus infection [31]. Despite this observation, non-secretors were present amongst rotavirus-positive cases, indicating that HBGA attachment may not be the only mechanism for rotavirus binding and subsequent entry. Early studies on rotavirus binding and entry described sialic acid as an attachment factor for some animal strains [32]. Alternative binding receptors such as sialic acid or yet unknown mechanisms could explain the presence of rotavirus infection in non-secretor individuals in our study. 

Studies have shown that rotavirus VP4 (VP8*) binds to HBGAs in a strain-specific manner [13]. Xu and colleagues showed that P[8] and P[4] rotavirus strains similarly bound to complex HBGAs via a ββ binding domain, while more distantly related P[6] strains bound simple H-type 1 structures in a βα binding domain [14]. In our study, a higher proportion of secretors was observed in P[8] (78%) and P[4] (76%) rotavirus infections compared to P[6] infections (53%). This suggested that secretors were significantly more susceptible to P[8] and P[4] strains than to P[6] strains (*p* < 0.01), while non-secretors were more likely to be infected with P[6] strains. These strain-specific interactions may also influence the circulation of rotavirus strains within the South African population, as observed in other settings [15,16,17].

A correlation in the prevalence of rotavirus VP4 strains and HBGA genotypes suggested that the circulation of rotavirus may be partially modulated by their ability to bind to host-defined HBGA receptors. Globally, G1P[8] is the predominantly circulating rotavirus genotype, with ~74% of global strains containing the P[8] VP4 strain [18]. However, studies have shown that rotavirus strains in Africa are more diverse, with P[8] comprising 32% of rotavirus cases, P[4] comprising 13% of rotavirus cases, and P[6] comprising 26% of rotavirus cases [18]. In South Africa, P[6] strains were detected in 25% of rotavirus cases between 2003 and 2006, and they continue to circulate [30,33]. In this study, the higher proportion of non-secretors (34%), naturally resistant to P[8] and P[4] rotavirus infections, may explain the 16% detection of P[6] strains [17,34]. The *FUT2* genetics of a population may define the availability of host HBGA receptors for rotavirus infection, which could drive the epidemiology of rotavirus strain circulation in a region. 

Discrepant results in Sanger sequencing revealed that five individuals were misclassified by RT-PCR as non-secretors (error rate 22.7%; 5/22), with sequencing identifying these five individuals as heterozygous secretors (Sese). The specimen sub-set comprised 58.5% secretors and 41.5% non-secretors based on RT-PCR genotyping, while the same specimens comprised 67.9% secretors and 32.1% non-secretors based on Sanger sequencing—an overall over-estimation of non-secretors of approximately 10%. This over-estimation of non-secretor genotypes is important to note for future studies, especially when using the TaqMan^®^ SNP Genotyping Assay targeting the G428A SNP in an African population where non-secretors are frequent. The proportion of non-secretors (34%) observed in our cohort of 500 individuals correlated with other studies in African populations where higher frequencies of non-secretors were observed [35,36]. 

Misclassification by the commercial genotyping assay was hypothesised to be due to a mutation noted ~50 bp downstream of the G428A SNP position. The manufacturer confirmed that the mutation affected the primer binding of the reverse primer to the functional copy of the *FUT2* gene in the five heterozygous secretors, resulting in the absence of PCR product for the FAM-labelled probe (which detects the presence of the allele without the G428A SNP) to bind. Interestingly, the mutation was found in 9% of African populations compared to 2% in all populations in the 1000 genomes project [37]. Sanger sequencing remains an important tool to investigate host genetic factors such as secretor status, and further sequencing will be considered to examine the extent of the *FUT2* G514R mutation detected in this study. 

Studies have indicated that secretor status can influence antibody titres to rotavirus [36], the incidence of gastrointestinal disease [38], and immune responses to rotavirus vaccines [28]. Rotarix^®^ and RotaTeq^®^ vaccines both contain P[8] vaccine constructs and require multiplication in intestinal cells to elicit local gut immunity [39,40]. The absence of HBGA attachment factors in non-secretors may reduce the replicative capacity of P[8] vaccine strains. The observation that non-secretors in Africa exhibit a natural resistance to wild-type P[8] strains may provide insights into the differences in vaccine efficacy across populations [5]. A study by Kazi and colleagues identified a link between the immune response to rotavirus P[8] vaccines and secretor status [28], and these associations have since been observed elsewhere [19,22,41]. Since patient sera were not collected as part of the RSSP, we could not investigate the direct effect of secretor status on rotavirus vaccine immune responses. Future studies investigating links between secretor status and variables such as vaccine immune responses, breastfeeding in young children, population genetics, and gut microbiome compositions, as well as alternative binding receptors for rotavirus entry, should be considered. 

The limitations of this study include the small sample size of P[6] rotavirus cases available for further analysis (16%; 40/250). A larger sample size of rotavirus genotypes would be beneficial in confirming the relationship between specific rotavirus VP4 strains and secretor status. Another limitation of this study was the discordant results between RT-PCR genotyping and Sanger sequencing, resulting in the misclassification of heterozygous secretors by RT-PCR. Only 13% (22/171) of non-secretor genes were sequenced due to budget constraints, and additional funding will be sought to expand the sequencing of the *FUT2* gene of non-secretors in South Africa. A final limitation of this study was not including analysis of the related *FUT3* Lewis genes as it may also impact susceptibility to rotavirus infections. Future studies should consider the genetics of a cohort before utilising genotyping techniques, since alternative SNPs may be present which may skew results. 

## 4. Materials and Methods 

The South African RSSP enrolled children under the age of five years hospitalised for diarrhoea at various sites across South Africa (Protocol M091018, approved by the Human Research Ethics Committee (Medical) of the University of Witwatersrand). Diarrhoea was defined as three or more loose stools in past 24 h, with or without vomiting. 

Informed consent was obtained from each child’s parent or guardian prior to participation in the RSSP. Stool and dried blood spot (DBS) specimens were collected from enrolled participants, and each child’s stool was screened as part of the RSSP for rotavirus group A (ProspecT™ Rotavirus Microplate Assay, Oxoid, Basingstoke, UK). Rotavirus-positive cases were genotyped using conventional RT-PCR methods and primers for G-specific and P-specific genotypes to determine the GxP[x] rotavirus strain [42]. 

This sub-study was conducted in accordance with the Declaration of Helsinki, and the project entitled “Investigation of secretor status, rotavirus VP4 genotypes, and gastrointestinal microbiomes in cases of diarrhoea in South Africa” (Protocol number 222/2018) was approved by the Research Ethics Committee, Faculty of Health Sciences, University of Pretoria, in May 2018. 

For this study, children enrolled in the RSSP between 2009 and 2017 with available DBS specimens were identified, and rotavirus-negative cases (n = 250) were randomly selected. Rotavirus GxP[x] genotypes were previously determined as part of the RSSP [30], and the rotavirus-positive subset (n = 250) was selected to represent the major rotavirus VP4 genotypes (P[8], P[4], and P[6]), with cases and controls selected randomly where possible. 

Secretor status was investigated using DBS specimens. DNA from DBS specimens was extracted using a QIAamp DNA Mini kit (Qiagen Inc., Valencia, CA, USA) according to the manufacturer’s instructions with one modification prior to extraction. The manufacturer’s protocol was modified to improve lysis by incubating DBS cards (~1 cm diameter) in a 200 µL buffer ATL overnight at 37 °C, instead of at 85 °C for 10 min. Following extraction, DNA was stored at −40 °C at the Centre for Enteric Diseases (Virology), National Institute for Communicable Diseases. 

Secretor status was determined by detecting the presence or absence of the *FUT2* G428A SNP using a Predesigned TaqMan^®^ SNP Genotyping assay (Life Technologies Corporation, CA, USA, supplied by Thermo Fisher Scientific, Carlsbad, CA, USA) in a 10 µL reaction volume according to the manufacturer’s instructions [27,43]. 

The Sanger sequencing of 10% of the cohort *FUT2* genes was performed to ensure that alternative non-secretor-causing SNPs, which may be undetected by this assay, were absent. The specimens were selected to include all secretor genotypes, with a slight selection bias towards heterozygous secretors (n = 19) and non-secretors (n = 22) compared to homozygous secretors (n = 12), as well as a range of cycle threshold values (Ct range of 10–39) obtained during RT-PCR. The coding exon 2 region of the *FUT2* gene was amplified using the FUT2Ex2F and FUT2Ex2R primers [7], cleaned using an ExoSAP-IT™ PCR Product Cleanup protocol (Thermo Fisher), and sequenced using a BigDye™ Terminator v3.1 Cycle Sequencing kit (Applied Biosystems, Life Technologies, Waltham, MA, USA) on an Applied Biosystems 3500xL Genetic Analyzer instrument (Applied Biosystems). Sequences were aligned to a *FUT2* protein-coding reference sequence (NG_007511.1:11987-13018 *Homo sapiens* fucosyltransferase 2 (*FUT2*), RefSeqGene on chromosome 19) (NCBI) using Molecular Evolutionary Genetics Analysis software version 7.0.26 (MEGA7). 

The sequences of the FUT2 exon 2 region of 10% of the cohort were submitted to BankIt (National Center for Biotechnology Information, Bethesda, MD, USA), and the accession numbers are as follows: MW036696, MW036697, MW036698, MW036699, MW036700, MW036701, MW036702, MW036703, MW036704, MW036705, MW036706, MW036707, MW036708, MW036709, MW036710, MW036711, MW036712, MW036713, MW036714, MW036715, MW036716, MW036717, MW036718, MW036719, MW036720, MW036721, MW036722, MW036723, MW036724, MW036725, MW036726, MW036727, MW036728, MW036729, MW036730, MW036731, MW036732, MW036733, MW036734, MW036735, MW036736, MW036737, MW036738, MW036739, MW036740, MW036741, MW036742, MW036743, MW036744, MW036745, MW036746, MW036747, MW036748.

Statistical analyses using Chi-squared tests and univariate logistic regression models were performed using STATA version 14.0, where *p* < 0.05 was considered significant (StataCorp College Station, TX, USA). 

## 5. Conclusions

Rotavirus susceptibility appeared to be influenced by secretor status in this study of South African children hospitalised with acute diarrhoea. Secretors expressing HBGAs in gut mucosal surfaces were more likely to be infected with rotavirus, specifically the P[8] and P[4] strains, compared to non-secretors. Non-secretors, with an absence of HBGAs in the gut, appeared to be less susceptible to rotavirus P[8] and P[4] infections compared to secretors—thus, the P[6] genotype was more frequent in these individuals. Interactions between rotavirus and secretor status could provide insights into the circulation of rotavirus strains amongst genetically diverse populations. Insights into the potential causes of altered rotavirus susceptibility and subsequent vaccine efficacy will aid in minimising the burden of disease. Diarrhoeal deaths are preventable, and secretor status may be an important host genetic factor to help understand and improve rotavirus disease prevention. Finally, the choice of assay for detecting or classifying secretor status in different populations should be carefully considered because the tools currently available all have pros and cons associated with their use. 

## Figures and Tables

**Table 1:** The distribution of secretors and non-secretors amongst VP4 genotypes P[8], P[4], and P[6] of rotavirus-positive cases (RV+; n = 250).

Rotavirus Genotypes:	P[8] Infections (n = 124)	P[4] Infections (n = 86)	P[6] Infections (n = 40)
Secretors	79% (98/124)	77% (66/86)	52.5% (21/40)
Non-secretors	21% (26/124)	23% (20/86)	47.5% (19/40)
*p*-values for each comparison	P[8] vs. P[4]: *p* = 0.693		
	P[8] vs. P[6]: *p* = 0.001		
	P[4] vs. P[6]: *p* = 0.006		
	P[8] + P[4] vs. P[6]: *p* = 0.001		

## Supplementary Materials

The following are available online at https://www.mdpi.com/2076-0817/9/10/795/s1, File: Final DBS Cohort Results_7.9.2020.

## References

[1] Troeger C., Forouzanfar M., Rao P.C., Khalil I., Brown A., Reiner R.C., Fullman N., Thompson R.L., Abajobir A., Ahmed M. (2017). Estimates of global, regional, and national morbidity, mortality, and aetiologies of diarrhoeal diseases: A systematic analysis for the Global Burden of Disease Study 2015. Lancet Infect. Dis..

[2] Troeger C., Khalil I.A., Rao P.C., Cao S., Blacker B.F., Ahmed T., Armah G., Bines J.E., Brewer T.G., Colombara D.V. (2018). Rotavirus vaccination and the global burden of rotavirus diarrhea among children younger than 5 years. JAMA Pediatr..

[3] Burnett E., Jonesteller C.L., Tate J.E., Yen C., Parashar U.D. (2017). Global impact of rotavirus vaccination on childhood hospitalizations and mortality from diarrhea. J. Infect. Dis..

[4] Steele A.D., Victor J.C., Carey M.E., Tate J.E., Atherly D.E., Pecenka C., Diaz Z., Parashar U.D., Kirkwood C.D. (2019). Experiences with rotavirus vaccines: Can we improve rotavirus vaccine impact in developing countries?. Hum. Vaccin. Immunother..

[5] Velasquez D.E., Parashar U., Jiang B. (2018). Decreased performance of live attenuated, oral rotavirus vaccines in low-income settings: Causes and contributing factors. Expert Rev. Vaccines.

[6] Parker E.P.K., Ramani S., Lopman B.A., Church J.A., Iturriza-Gómara M., Prendergast A.J., Grassly N.C. (2018). Causes of impaired oral vaccine efficacy in developing countries. Future Microbiol..

[7] Ferrer-Admetlla A., Sikora M., Laayouni H., Esteve A., Roubinet F., Blancher A., Calafell F., Bertranpetit J., Casals F. (2009). A natural history of FUT2 polymorphism in humans. Mol. Biol. Evol..

[8] Ramani S., Hu L., Venkataram Prasad B.V., Estes M.K. (2016). Diversity in rotavirus-host glycan interactions: A “sweet” spectrum. Cell. Mol. Gastroenterol. Hepatol..

[9] Monedero V., Buesa J., Rodríguez-Díaz J. (2018). The interactions between host glycobiology, bacterial microbiota, and viruses in the gut. Viruses.

[10] Van Trang N., Vu H.T., Le N.T., Huang P., Jiang X., Anh D.D. (2014). Association between norovirus and rotavirus infection and histo-blood group antigen types in vietnamese children. J. Clin. Microbiol..

[11] Nordgren J., Sharma S., Kambhampati A., Lopman B., Svensson L. (2016). Innate Resistance and Susceptibility to Norovirus Infection. PLoS Pathog..

[12] Nordgren J., Svensson L. (2019). Genetic susceptibility to human norovirus infection: An update. Viruses.

[13] Zhang X.F., Long Y., Tan M., Zhang T., Huang Q., Jiang X., Tan W.F., Li J.D., Hu G.F., Tang S. (2016). P[8] and P[4] rotavirus infection associated with secretor phenotypes among children in south China. Sci. Rep..

[14] Xu S., Liu Y., Tan M., Zhong W., Zhao D., Jiang X., Michael A. (2019). Molecular basis of P[6] and P[8] major human rotavirus VP8* domain interactions with histo-blood group antigens. BioRxiv.

[15] Heylen E., Zeller M., Ciarlet M., Lawrence J., Steele D., Van Ranst M., Matthijnssens J. (2016). Human P[6] rotaviruses from sub-saharan Africa and southeast Asia are closely related to those of human P[4] and P[8] rotaviruses circulating worldwide. J. Infect. Dis..

[16] Gozalbo-Rovira R., Ciges-Tomas J.R., Vila-Vicent S., Buesa J., Santiso-Bellón C., Monedero V., Yebra M.J., Marina A., Rodríguez-Díaz J. (2019). Unraveling the role of the secretor antigen in human rotavirus attachment to histo-blood group antigens. PLOS Pathog..

[17] Hu L., Sankaran B., Laucirica D.R., Patil K., Salmen W., Ferreon A.C.M., Tsoi P.S., Lasanajak Y., Smith D.F., Ramani S. (2018). Glycan recognition in globally dominant human rotaviruses. Nat. Commun..

[18] Todd S., Page N.A., Duncan Steele A., Peenze I., Cunliffe N.A. (2010). Rotavirus strain types circulating in Africa: Review of studies published during 1997–2006. J. Infect. Dis..

[19] Armah G.E., Cortese M.M., Dennis F.E., Yu Y., Morrow A.L., McNeal M.M., Lewis K.D.C., Awuni D.A., Armachie J., Parashar U.D. (2019). Rotavirus vaccine take in infants is associated with secretor status. J. Infect. Dis..

[20] Sharma S., Hagbom M., Svensson L., Nordgren J. (2020). The Impact of Human Genetic Polymorphisms on Rotavirus Susceptibility, Epidemiology, and Vaccine Take. Viruses.

[21] Bucardo F., Reyes Y., Rönnelid Y., González F., Sharma S., Svensson L., Nordgren J. (2019). Histo-blood group antigens and rotavirus vaccine shedding in Nicaraguan infants. Sci. Rep..

[22] Pollock L.E. (2018). Predictors of Vaccine Viral Replication, Immune Response and Clinical Protection Following Oral Rotavirus Vaccination in Malawian Children. Ph.D. Thesis.

[23] Clarke E., Desselberger U. (2015). Correlates of protection against human rotavirus disease and the factors influencing protection in low-income settings. Mucosal Immunol..

[24] Wannhoff A., Folseraas T., Brune M., Rupp C., Friedrich K., Knierim J., Weiss K.H., Sauer P., Flechtenmacher C., Schirmacher P. (2016). A common genetic variant of fucosyltransferase 2 correlates with serum carcinoembryonic antigen levels and affects cancer screening in patients with primary sclerosing cholangitis. United Eur. Gastroenterol. J..

[25] Colston J.M., Francois R., Pisanic N., Peñataro Yori P., McCormick B.J.J., Olortegui M.P., Gazi M.A., Svensen E., Ahmed M.M.M., Mduma E. (2019). Effects of child and maternal histo-blood group antigen status on symptomatic and asymptomatic enteric infections in early childhood. J. Infect. Dis..

[26] Günaydin G., Nordgren J., Sharma S., Hammarstrom L. (2016). Association of elevated rotavirus-specific antibody titers with HBGA secretor status in Swedish individuals: The FUT2 gene as a putative susceptibility determinant for infection. Virus Res..

[27] Bucardo F., Nordgren J., Reyes Y., Gonzalez F., Sharma S., Svensson L. (2018). The Lewis A phenotype is a restriction factor for Rotateq and Rotarix vaccine-take in Nicaraguan children. Sci. Rep..

[28] Kazi A.M., Cortese M.M., Yu Y., Lopman B., Morrow A.L., Fleming J.A., McNeal M.M., Steele A.D., Parashar U.D., Zaidi A.K.M. (2017). Secretor and salivary ABO blood group antigen status predict rotavirus vaccine take in infants. J. Infect. Dis..

[29] GERMS-SA Annual Report 2017. http://www.nicd.ac.za/index.php/publications/germs-annual-reports/.

[30] Page N.A., Seheri L.M., Groome M.J., Moyes J., Walaza S., Mphahlele J., Kahn K., Kapongo C.N., Zar H.J., Tempia S. (2018). Temporal association of rotavirus vaccination and genotype circulation in South Africa: Observations from 2002 to 2014. Vaccine.

[31] De Mattos L.C. (2016). Structural diversity and biological importance of ABO, H, Lewis and secretor histo-blood group carbohydrates. Brazilian J. Hematol. Hemotherapy.

[32] Isa P., Arias C.F., López S. (2006). Role of sialic acids in rotavirus infection. Glycoconj. J..

[33] Seheri L.M., Page N., Dewar J.B., Geyer A., Nemarude A.L., Bos P., Esona M., Steele A.D. (2010). Characterization and Molecular Epidemiology of Rotavirus Strains Recovered in Northern Pretoria, South Africa during 2003–2006. J. Infect. Dis..

[34] Mwenda J.M., Ntoto K.M., Abebe A., Enweronu-laryea C., Amina I., Mchomvu J., Kisakye A., Mpabalwani E.M., Pazvakavambwa I., Armah G.E. (2018). Burden and Epidemiology of Rotavirus Diarrhea in Selected African Countries: Preliminary Results from the African Rotavirus Surveillance Network. J. Infect. Dis..

[35] Thorne L., Nalwoga A., Mentzer A.J., De Rougemont A., Hosmillo M., Webb E., Nampiija M., Muhwezi A., Carstensen T., Gurdasani D. (2018). The first norovirus longitudinal seroepidemiological study from sub-Saharan Africa reveals high seroprevalence of diverse genotypes associated with host susceptibility factors. J. Infect. Dis..

[36] Nordgren J., Sharma S., Bucardo F., Nasir W., Günaydin G., Ouermi D., Nitiema L.W., Becker-Dreps S., Simpore J., Hammarström L. (2014). Both lewis and secretor status mediate susceptibility to rotavirus infections in a rotavirus genotype-dependent manner. Clin. Infect. Dis..

[37] Auton A., Abecasis G.R., Altshuler D.M., Durbin R.M., Abecasis G.R., Bentley D.R., Chakravarti A., Clark A.G., Donnelly P., Eichler E.E. (2015). A global reference for human genetic variation. Nature.

[38] Payne D.C., Currier R.L., Staat M.A., Sahni L.C., Selvarangan R., Halasa N.B., Englund J.A., Weinberg G.A., Boom J.A., Szilagyi P.G. (2015). Epidemiologic association between FUT2 secretor status and severe rotavirus gastroenteritis in children in the United States. JAMA Pediatr..

[39] Yen C., Healy K., Tate J.E., Parashar U.D., Bines J., Neuzil K., Santosham M., Steele A.D. (2016). Rotavirus vaccination and intussusception—Science, surveillance, and safety: A review of evidence and recommendations for future research priorities in low and middle income countries. Hum. Vaccines Immunother..

[40] Schellack N., Naested C., Schellack G., Meyer H., Motubatse J., Mametja K., Makola F. (2017). Schellack_2017_The management of rotavirus disease in children. SA Pharm. J..

[41] Lee B., Dickson D.M., DeCamp A.C., Ross Colgate E., Diehl S.A., Uddin M.I., Sharmin S., Islam S., Bhuiyan T.R., Alam M. (2018). Histo–blood group antigen phenotype determines susceptibility to genotype-specific rotavirus infections and impacts measures of rotavirus vaccine efficacy. J. Infect. Dis..

[42] World Health Organization (WHO) (2009). Manual of Rotavirus Detection and Characterization Methods.

[43] Thermo Fisher Scientific (2017). TaqMan ® SNP Genotyping Assays: User Guide.

